# Rational design and synthesis of novel phenylsulfonyl-benzamides as anti-prostate cancer agents†
†The authors declare no competing interests.
‡
‡Electronic supplementary information (ESI) available. See DOI: 10.1039/c7md00164a
§
§This work is dedicated to the memory of Prof. Chris McGuigan, a great colleague and scientist, invaluable source of inspiration and love for research.


**DOI:** 10.1039/c7md00164a

**Published:** 2017-05-26

**Authors:** Marcella Bassetto, Salvatore Ferla, Gilda Giancotti, Fabrizio Pertusati, Andrew D. Westwell, Andrea Brancale, Christopher McGuigan

**Affiliations:** a School of Pharmacy and Pharmaceutical Sciences , Redwood Building, King Edward VII Avenue , CF10 3NB , Cardiff , Wales , UK . Email: BassettoM@cardiff.ac.uk

## Abstract

Prostate cancer is a major cause of male death worldwide and the identification of new efficient treatments is constantly needed. Different non-steroidal androgen receptor antagonists are approved also in the case of castration-resistant cancer forms. Using a rational approach and molecular modelling studies to modify the structure of antiandrogen drug bicalutamide, a new series of phenylsulfonyl-benzamide derivatives was designed and synthesised. Their antiproliferative activities were evaluated in four different human prostate cancer cell lines and several new compounds showed significantly improved IC_50_ values in the low μM range. The cytotoxicity profile was also evaluated for the novel structures in the HEK293 cell line.

## Abstract 2

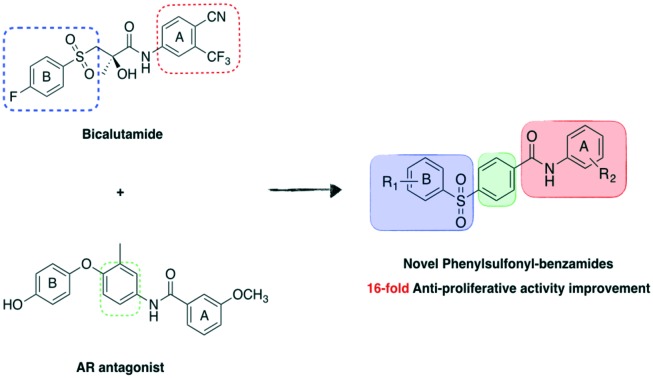
A novel antiproliferative molecular scaffold was designed by rational modification of known antiandrogens, achieving a significant improvement in anti-cancer activity.

## Introduction

**Fig. 1:**
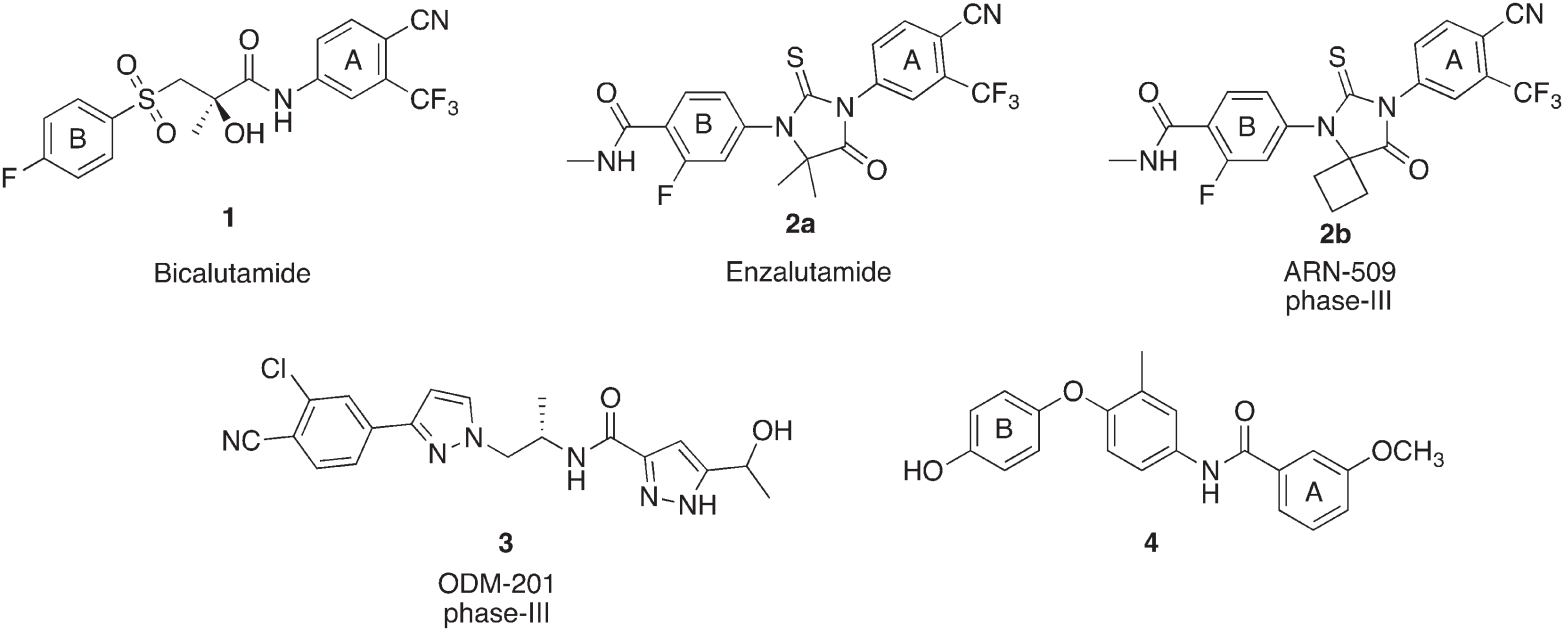
Structure of anti-androgen small molecules approved by FDA (**1** and **2a**), in clinical development for the treatment of PC (**2b** and **3**) or in early-stage development (**4**). Two differently substituted aromatic rings, A and B, are connected by a linker.

Prostate cancer (PC) is one of the major causes of male death worldwide, representing the second most common cancer in males.^1^ One of the main treatment strategies against this disease is currently represented by androgen deprivation:^2^ bicalutamide (**1**) and enzalutamide (**2**) are two non-steroidal androgen receptor (AR) antagonist drugs approved to treat PC (Fig. 1). Unfortunately, these anti-androgens tend to become ineffective in a few years' time due to adaptive mutations on the structure of the androgen receptor, which switch the function of these drugs from antagonist to agonist.^2^

One of the most common mutations found for bicalutamide is W741L in helix 12 of the receptor,^3^ which allows the protein to adopt its closed agonist conformation even in the presence of the antagonist: with this mutation, due to some residual structural flexibility in **1**, ring B can bend to occupy an inner portion of the ligand-binding domain, thus allowing the closure of the receptor into its agonist conformation. Treatment with enzalutamide induces instead a F876L mutation in the AR, which also confers an antagonist to agonist switch in activity for the drug.^4^ Second-generation antiandrogen ARN-509 (Fig. 1), which is now in Phase III clinical trials,^5^ is also associated with the insurgence of resistance through the F876L mutation,^6^ while new-generation AR inhibitor ODM-201 (Fig. 1), now in Phase III trials, has shown retained activity in the F876L AR mutant.^7^

Most non-steroidal antiandrogens are structurally characterised by two differently substituted aromatic rings, named ring A and ring B, connected by a linker, either linear (bicalutamide-like compounds) or cyclic (enzalutamide-like compounds) (Fig. 1).^8^ Recently, a novel 4-(4-benzoylaminophenoxy) phenol antiandrogen scaffold (**4**), derived from the natural pigment curcumin, has been reported, in which a central phenyl group is acting as linker connecting two different aromatic rings.^9^

**Fig. 2:**
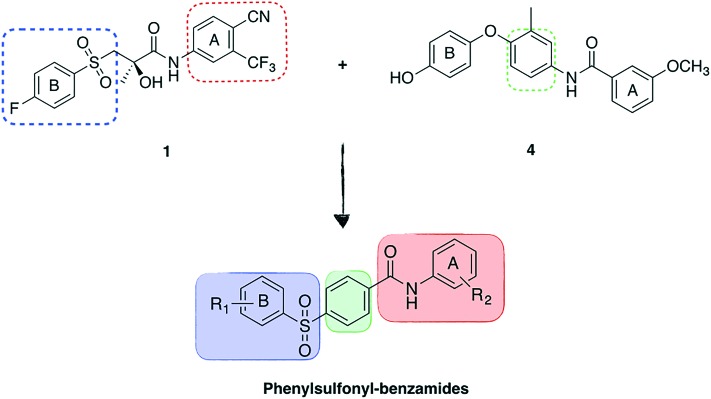
Rational design of novel phenylsulfonyl-benzamide scaffold.

With the aim to rigidify the structure of bicalutamide and obtain new anticancer agents, we designed a novel molecular scaffold in which we replaced the flexible bicalutamide methyl-hydroxy-methylene linker with a rigid phenyl group, maintaining the two lateral aromatic rings commonly found in antiandrogen compounds (Fig. 2). Moreover, following an approach previously proven successful in our research group for increasing bicalutamide and enzalutamide anti-prostate cancer activity,^10^,^11^ different perfluoro groups were systematically inserted in aromatic ring B (R_2_ substituent), replacing the classical 4-cyano substituent. The phenolic 4-hydroxy group of **4**, previously reported as essential for AR antagonistic activity,^9^ was either maintained, in order to evaluate its importance for anti-prostate cancer effect, or replaced with a 3-trifluoromethyl group, as a means to incorporate and evaluate one of the most successful modifications we have previously found in other series of related compounds.^10^,^11^

Following this approach, a novel family of phenylsulfonyl-benzamides was developed (Fig. 2), and their potential anti prostate cancer activity was evaluated in four different human prostate cancer cell lines, together with their cytotoxicity in the HEK293 cell line.

## Results and discussion

### Molecular modelling

**Fig. 3:**
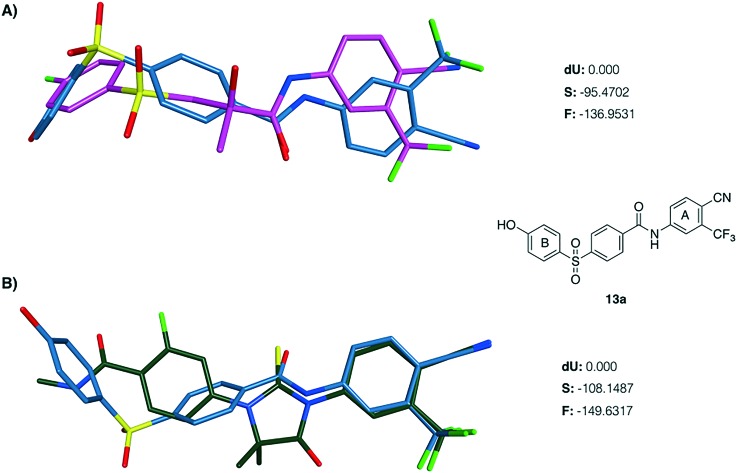
Flexible alignment between **13a** (carbon atoms in light blue) with the predicted active binding conformations of bicalutamide (A-carbon atoms in purple) and enzalutamide (B-carbon atoms in dark green).

A series of flexible alignment and molecular docking studies was performed to evaluate the proposed structural modifications. For these analyses, compound **13a**, which maintains bicalutamide and enzalutamide aromatic substituents in ring A and is characterised by the fully oxidised sulfonyl linker, was chosen as representative for the novel series of compounds. In a previous work,^10^ we have extensively discussed and described the most likely binding mode of bicalutamide and enzalutamide in the homology model of the androgen receptor (AR) open antagonist conformation. In the present study, the predicted active conformation for the two drugs was kept rigid and a flexible alignment with newly designed compound **13a** was performed using MOE2015.10,^12^ in order to evaluate its potential structural overlapping with the two active conformations. **13a** aligns well with the binding pose of the parent compounds, with the newly introduced phenyl ring conferring the desired structural rigidity to lock the molecule in the active conformation (Fig. 3). Moreover, as reported in the ESI‡ (Table S1), the two conformations obtained for **13a** show a large negative similarity score *F*, meaning that **13a** shows a high shape and functional similarity to the two parent compounds. Finally, the d*U* value found for both alignments is lower than 1 kcal mol^–1^, meaning that the obtained conformations are energetically favoured. The better (more negative) similarity score obtained for the alignment with enzalutamide is a further confirmation that the introduction of the phenyl linker rigidifies the novel structure, potentially locking the molecule in an active conformation.

**Fig. 4:**
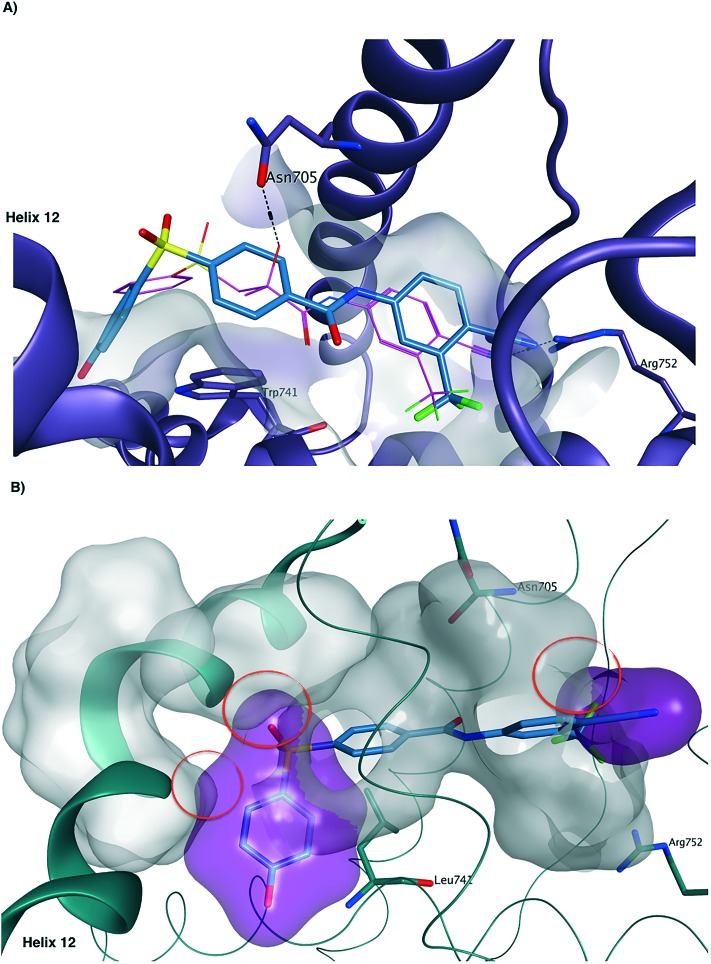
A) Predicted binding mode of **13a** (carbon atoms in light blue) in the AR homology model.^10^ The compound occupies the LBD in a similar way to bicalutamide (carbon atoms in purple). B) **13a** in the bicalutamide-W741I AR closed conformation crystal structure ; 1Z95 (B).^3^ The compound shows major clashes (purple surface), highlighted by red circles, with the protein surface (in grey) of the closed AR structure, especially with helix 12, indicating that it might impede the AR closure even in the presence of adaptive mutations.

Fig. 4A shows the docking results found for **13a** in the ligand binding domain (LBD) of the open AR homology model, obtained using Plants docking software.^13^ The extra phenyl ring, as observed in the flexible alignment results, maintains the molecule in a rigid extended conformation that entirely occupies the binding site, similarly to bicalutamide and enzalutamide. The phenolic hydroxyl group is facing outside the binding groove, therefore its replacement with other functions is predicted to be tolerated. Docking results for **13a** in the bicalutamide-resistant AR mutant W741L crystal structure (PDB ID ; 1Z95),^3^ which corresponds to the closed active conformation of the receptor, indicate that, due to its increased rigidity, the novel molecule cannot fit the AR antagonist conformation, as highlighted by the steric clashes between **13a** and helix 12 (Fig. 4B). These results suggest that the novel scaffold could maintain its antagonist activity also in the presence of the W741L mutation.

### Chemistry

**Scheme 1:**
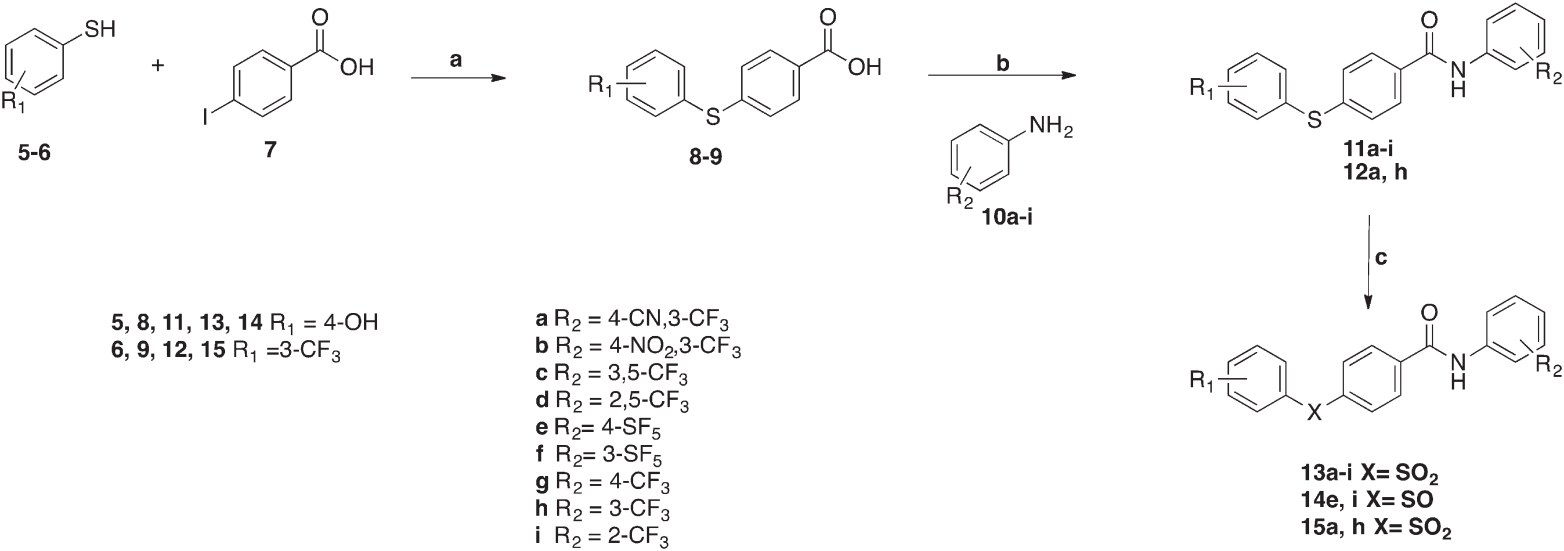
Synthetic pathway for the synthesis of phenylsulfonyl-benzamides analogues. Reagents and conditions: (a) KOH, Cu, H_2_O, reflux, 24 h; (b) **8–9** (1 equiv.), SOCl_2_ (1.2 equiv.), DMA, –15 to –10 °C to rt. After 1 h **10a–i** (1 equiv.) dissolved in DMA is added, rt, on; (c) mCPBA (1.4 equiv.), DCM, rt, on.

Scheme 1 reports the three-step synthetic pathway developed and optimised for the preparation of the newly designed compounds.

In the first step a copper catalysed C–S coupling reaction was used for the preparation of intermediates **8–9**. The conditions for this step were optimised from a reported procedure.^14^ After 12 hours reflux as in the published protocol, 4-iodobenzoic acid **7** was still present in the reaction mixture. No difference could be observed with a prolonged reaction time, while total consumption of starting material **7** was finally achieved by doubling the equivalents of thiophenol and base. The new conditions were applied to react differently substituted thiophenols **5–6** with 4-iodobenzoic acid **7** for 24 hours in water, giving the desired different substituted phenylthio-benzoic acids in an almost quantitative yield. Amides **11a–i** and **12a**, **h** were obtained reacting the corresponding anilines **10a–i** with **8** and **9** in the presence of thionyl chloride in DMA, modifying a literature procedure.^15^ In the last step, sulphur derivatives **11a–i** and **12a**, **h** were oxidized to the corresponding sulfones **13a–i** and **15a**, **h** using mCPBA at 25 °C.^10^ In two cases, the corresponding product of sulphur partial oxidation was also isolated (**14e** and **14i**). These two sulfoxides were characterised by low solubility in the reaction solvent, therefore complete oxidation to the corresponding sulfone was much slower and did not go to completion within the reaction time. Using this synthetic approach, 24 novel phenylsulfonyl-benzamides derivatives were prepared, purified and fully characterised.

Standard bicalutamide and enzalutamide were also prepared following reported procedures.^10^

### *In vitro* 2D monolayer antiproliferative assay

All new compounds along with the two standards were tested for their ability to inhibit survival and/or cell proliferation in four human prostate cancer cell lines: LNCaP, 22Rv1, VCaP, androgen-sensitive cell lines, and DU145, a non hormone-sensitive cell line. Table 1 shows the antiproliferative results for each single cell line (absolute IC_50_ in μM), along with the overall effect in the four cell lines reported as geometric mean. Activity found for bicalutamide and enzalutamide is consistent with previously published data for some of these specific cell lines,^16^–^18^ confirming the reliability of the assay.

**Table 1:** Antiproliferative activity for the novel phenylsulfonyl-benzamide derivatives. All data are mean values from triplicate experiments, with standard deviations of ±10% of the value quoted unless otherwise stated. Compound **15h** showed solubility issues

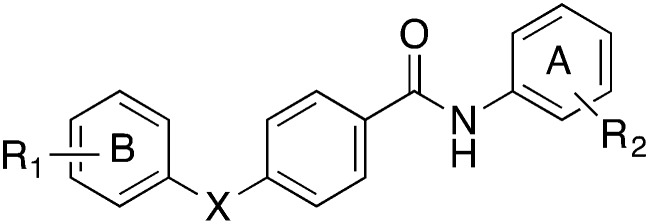								
Compound	R_1_	X	R_2_	Absolute IC_50_ (μM)				
				22Rv1	DU-145	LNCaP	VCaP	Geo. mean
**Bicalutamide**	—	—	—	49.58	49.20	45.27	68.37	52.42
**Enzalutamide**	—	—	—	24.77	44.55	20.90	24.47	27.41
**11a**	4-OH	S	4-CN, 3-CF_3_	6.24	7.77	5.43	9.55	7.08
**11b**	4-OH	S	4-NO_2_, 3-CF_3_	4.50	5.74	3.58	5.19	4.68
**11c**	4-OH	S	3,5-CF_3_	**3.35**	**5.60**	**2.39**	**2.59**	**3.28**
**11d**	4-OH	S	2,5-CF_3_	10.34	16.63	11.42	10.88	12.09
**11e**	4-OH	S	4-SF_5_	3.71	6.76	4.86	7.15	5.43
**11f**	4-OH	S	3-SF_5_	2.94	4.35	3.32	6.10	4.01
**11g**	4-OH	S	4-CF_3_	6.23	8.55	8.30	9.68	8.09
**11h**	4-OH	S	3-CF_3_	5.73	5.95	9.66	10.58	7.69
**11i**	4-OH	S	2-CF_3_	30.92	35.68	11.43	32.27	25.25
**12a**	3-CF_3_	S	4-CN, 3-CF_3_	3.57	5.12	8.87	5.66	5.50
**12h**	3-CF_3_	S	3-CF_3_	6.96	9.89	12.47	28.22	12.47
**13a**	4-OH	SO_2_	4-CN, 3-CF_3_	12.08	12.62	4.68	8.56	8.84
**13b**	4-OH	SO_2_	4-NO_2_, 3-CF_3_	7.30	8.85	4.96	8.57	7.24
**13c**	4-OH	SO_2_	3,5-CF_3_	6.44	9.92	8.41	9.72	8.50
**13d**	4-OH	SO_2_	2,5-CF_3_	20.66	27.79	23.18	30.48	25.24
**13e**	4-OH	SO_2_	4-SF_5_	5.78	10.85	2.49	5.35	5.37
**14e**	4-OH	SO	4-SF_5_	**5.60**	**11.02**	**0.90**	**3.53**	**3.75**
**13f**	4-OH	SO_2_	3-SF_5_	5.66	6.01	3.98	10.81	6.18
**13g**	4-OH	SO_2_	4-CF_3_	16.79	30.69	10.75	27.16	19.69
**13h**	4-OH	SO_2_	3-CF_3_	15.06	17.73	10.19	30.08	16.84
**13i**	4-OH	SO_2_	2-CF_3_	49.31	45.88	39.31	65.20	49.07
**14i**	4-OH	SO	2-CF_3_	100.00	100.00	61.98	97.60	88.19
**15a**	3-CF_3_	SO_2_	4-CN, 3-CF_3_	3.57	4.70	3.73	7.87	4.71
**15h**	3-CF_3_	SO_2_	3-CF_3_	n.d	n.d	n.d	n.d	n.d

Considering their overall profile in the four cell lines (geometric mean), almost all the new derivatives performed significantly better than bicalutamide, improving its effect up to 16-fold. The new inhibitors showed concentration-dependent activity with mean IC_50_ values ranging from 3.2 μM to >100 μM. Compared with standard enzalutamide, antiproliferative effect was improved up to 8-fold.

Substituent R_1_ in ring B does not seem to influence the anticancer profile of the scaffold, as no substantial differences were found between the OH group in the *para* position or the CF_3_ in *meta* (*i.e.***11a***vs.***12a**). This finding is in accordance with the proposed binding mode for **13a**, in which the R_1_ substituent is facing outside the AR binding domain without making specific interactions.

In both our previous work on bicalutamide derivatives and in another published work on analogues of SARMs (selective androgen receptor modulators),^10^,^16^ thioether compounds (X = S) are associated with better antiproliferative activities than the corresponding sulfones (X = SO_2_), with a decrease of effect in the sulfones up to 8-fold. In the new scaffold presented in this work, no particular differences can be observed between thioether and sulfone compounds, with retained activity in most sulfones (*i.e.***11a***vs.***13a**) and only a small increase of IC_50_ values up to 2-fold in few cases (*i.e.***11c***vs.***13c**). The partially oxidized derivatives (X = SO) retain the effect of the corresponding thioethers and sulfones (*i.e.***11e** and **13e***vs.***14e**).

Removal of the CN or the NO_2_ group in the *para* position of ring B is associated with retained activity, as found for mono 3-CF_3_ derivative **11h**. Moving the trifluoromethyl group from *meta* to *para* position does not have a significant effect on activity (*i.e.***11h***vs.***14g**), whereas the *ortho* substitution is characterized by a 3-fold reduction of overall effect in the four cell lines (*i.e.***11h***vs.***11i**). Replacing the trifluoromethyl group with a bigger –SF_5_ moiety in *para* or *meta* position is associated with a mildly improved activity profile (*i.e.***11g***vs.***14e**).

Addition of an extra CF_3_ in *meta* (R_2_ = 3,5-CF_3_), gives the best results in terms of overall activity in the four cell lines (*i.e.***11a** and **13h***vs.***14c**), as we had similarly observed in our previous research efforts.^10^ Also in this case, moving one trifluoromethyl group from *meta* to *ortho* position is associated with activity reduction (*i.e.***11c***vs.***11d**).

Examining each individual cell line, most of the new compounds showed the best results in the cell lines that highly express the androgen receptor. Among them, LNCaP cells were found to be the most sensitive to the new derivatives, with different compounds active at low μM concentrations and one reaching a sub μM IC_50_ value (**14e**). These data suggest that the newly prepared compounds are likely to retain an antagonistic effect on the androgen receptor. An interesting activity profile was also found in the DU-145 cell line (the least sensitive to our new molecules), which expresses low levels of the androgen receptor^19^ and is insensitive to androgen activity. The same effect can be observed also for parent bicalutamide (similar IC_50_ values across the four cell lines), suggesting that besides its canonical anti-androgen receptor action, a different antiproliferative mechanism could also be involved. The new phenylsulfonyl-benzamides, in addition to potentially increase the antagonistic activity on the AR, might also enhance the potential off target effect, even though in some cases they seem more selective for androgen-sensitive cell lines than bicalutamide (*i.e.***13e**, **14e**, **13g**). As a general observation, the improved antiproliferative activity of the novel compounds should be regarded as the result of the whole structure of each single derivative, since the data found appear to be a combination of the simultaneous effects of the substituent in ring B and linker X.

### Cytotoxicity assay

**Fig. 5:**
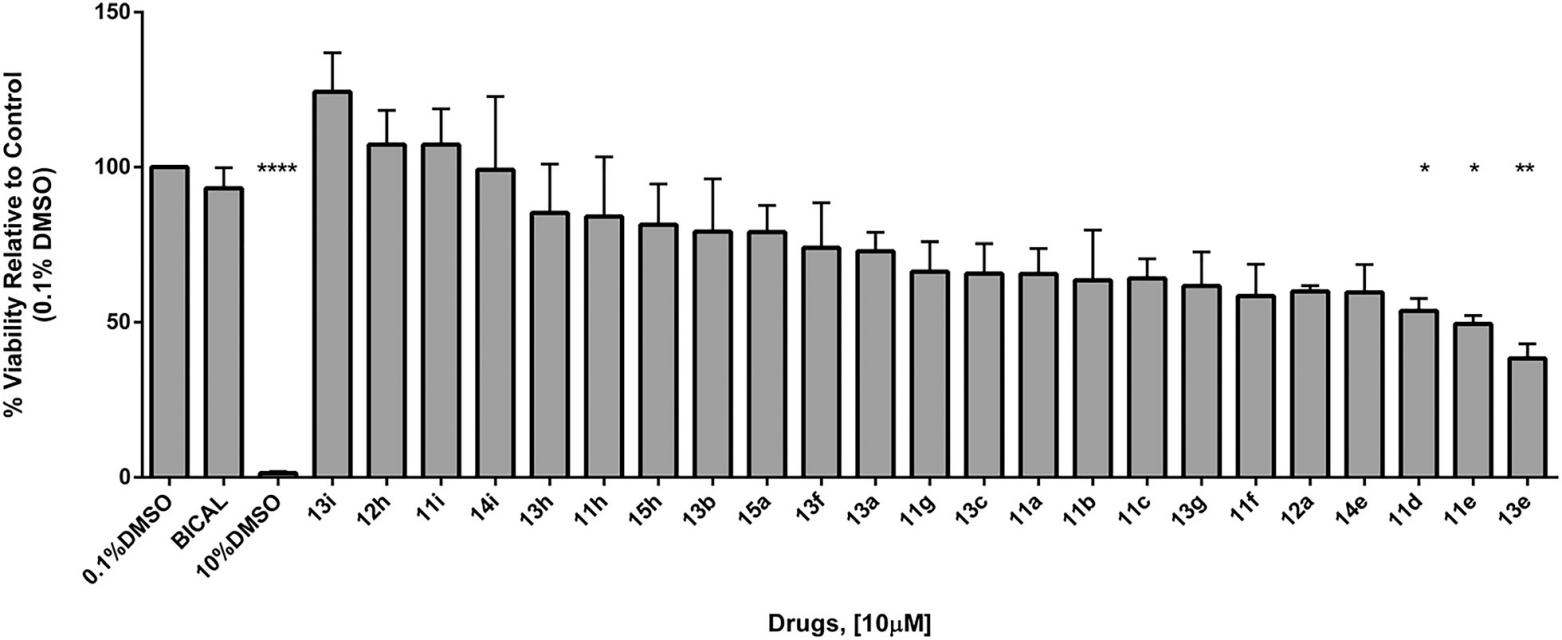
HEK293 human embryonic kidney cells were treated at 70% confluence with negative controls (0.1% DMSO, BICAL at 10 μM), positive control (10% DMSO) and compounds at 10 μM concentration for 24 hours. 20 μl of Cell Titer Blue reagent (Promega) was added to each well containing 100 μl media and cell were incubated for 2 h at 37 °C 5% CO_2_. Fluorescence was measured at 560/590 nm using a CLARIOstar luminescence plate reader (BMG Labtech). *N* = 4, *n* = 6 for controls, *n* = 3 for drugs. Errors are calculated as standard error of the mean. * = *p*-value < 0.05, ** = *p*-value < 0.01, *** = *p*-value < 0.001 **** = *p*-value < 0.0001 between bicalutamide and compounds.

A Cell Titer Blue viability assay was performed in the human embryonic kidney cell line HEK293, in order to evaluate the cytotoxicity profile of the newly prepared compounds. Fig. 5 shows the reduction of cell viability caused by the novel analogues and standard bicalutamide at a fixed concentration of 10 μM. Interestingly, as reported in the graph, only three compounds (**11d**, **11e** and **13e**) significantly reduced cell viability if compared to bicalutamide (statistical significance), and only one of them (**13e**) killed more than 50% of cells. Due to their higher cytotoxicity in comparison with bicalutamide, the antiproliferative effect of these three compounds in the prostate cancer cell lines could also be a consequence of their intrinsic toxicity, besides the canonical AR antagonist activity. Comparing the rest of the compounds with bicalutamide, even if some of them are characterized by an increased cytotoxicity, cell viability reduction is not statistically significant and less than 40% in most cases, therefore the observed cytotoxicity is comparable to the one found for bicalutamide. Moreover, it is worth to note that bicalutamide reaches its antiproliferative IC_50_ at 50 μM, therefore it would not show reduction of cell viability at 10 μM. On the contrary, most of our new compounds showed antiproliferative activity in the four prostate cancer cell lines at a low μM concentration (<10 μM), and even if some of them show a reduction of cell viability by 35–40%, their anticancer therapeutic window is still wide enough for consideration as a potential treatment for prostate cancer. In particular, compounds **11h**, **12h**, **13b** and **13h** are associated with a promising antiproliferative/cytotoxicity profile, with an antiproliferative effect much better than bicalutamide (<16 μM), and with the same cytotoxicity of bicalutamide at 10 μM (none or minimal reduction of cell viability).

## Conclusions

A novel family of phenylsulfonyl-benzamides was designed and synthesised after rational modification of the structure of antiandrogen drug bicalutamide, and many of the novel derivatives prepared show improved anti-prostate cancer properties. In particular, most of the new compounds display significantly improved anticancer activities in four different prostate cancer cell lines, with a cell viability reduction effect comparable to the one found for bicalutamide. Differently from previously reported SARMs, no significant difference in antiproliferative activity has been observed between our novel thioether and sulfone derivatives, representing a promising feature for future metabolic stability considerations. Within the 24 novel analogues evaluated, **11h**, **12h**, **13b** and **13h** have been selected as candidates for further investigations, due to their improved anticancer properties and their cytotoxicity profiles. With the aim to identify a pre-clinical candidate, these compounds will be studied for their binding to the AR, their potential off target effect and their pharmacokinetic properties.

## Experimental

All chemistry, biology and molecular modelling experimental procedures, along with compound characterisation, are fully described in the ESI.‡ All final compounds were purified by column chromatography or recrystallization and fully characterised by NMR (^1^H, ^13^C, ^19^F) and LRMS. All final compounds were found to be >95% pure by HPLC.

## Supplementary Material

Supplementary information
