# Supplementary material for: Rational design and synthesis of novel phenylsulfonyl-benzamides as anti-prostate cancer agents
Source: Medchemcomm. 2017 May 26;8(7):1414–20. doi: 10.1039/c7md00164a (PMC6072516; doi:10.1039/c7md00164a)
Supplement: Supplementary file 1 [file MD-008-C7MD00164A-s001.pdf]

Electronic Supplementary Material (ESI) for MedChemComm.
This journal is © The Royal Society of Chemistry 2017

Rational design and synthesis of novel phenylsulfonyl-benzamides as anti prostate cancer agents†‡

Marcella Bassetto[a]*, Salvatore Ferla[a]*[1], Gilda Giancotti[a] , Fabrizio Pertusati[a], Andrew D. Westwell[a], Andrea Brancale[a], Christopher McGuigan[a]

Supporting Information

1.1 General Chemistry methods

All solvents and reagents were used as obtained from commercial sources unless otherwise indicated. All reactions were performed under a nitrogen atmosphere. ^{1}H and ^{13}C NMR spectra were recorded with a Bruker Avance DPX500 spectrometer operating at 500 MHz for ^{1}H and 125 MHz for ^{13}C, with Me_4Si as internal standard. Deuterated chloroform was used as the solvent for NMR experiments, unless otherwise stated. ^{1}H chemical shifts values (δ) are referenced to the residual non-deuterated components of the NMR solvents (δ = 7.26 ppm for $CHCl_3$, etc.). The ^{13}C chemical shifts (δ) are referenced to $CDCl_3$ (central peak, δ = 77.0 ppm). Mass spectra were measured in positive or negative mode electrospray ionization (ESI). TLC was performed on silica gel 60 F254 plastic sheets. Column chromatography was performed using silica gel 60A (35–75 mesh, Fisher) or on an Isolera Biotage system. Purity of the newly prepared compounds was determined by NMR and HPLC-UV analysis (Thermo HPLC connected with UV detector). All compounds tested in biological assays were >95% pure. The purity of all final compounds was determined to be >95% by HPLC using the eluents water (eluent A), acetonitrile (eluent B) and methanol (eluent C) at the following conditions: Varian Pursuit, 150 mm × 4.6 mm, 5.0 µm, 1.0 mL/min, gradient 30 min 10% → 100% eluent B in eluent A (method 1) or gradient 30 min 10% → 100% eluent C in eluent A (method 2). Purity of intermediates was >90%, unless otherwise stated.

---

[a] School of Pharmacy and Pharmaceutical Sciences, Redwood Building, King Edward VII Avenue, CF10 3NB, Cardiff, Wales, UK E-mail: BassettoM@cardiff.ac.uk (Dr. M. Bassetto)

†The authors declare no competing interests.

‡Electronic supplementary information (ESI) available.

* These authors contributed equally to this work

This work is dedicated to the memory of Prof. Chris McGuigan, a great colleague and scientist, invaluable source of inspiration and love for research.

1.1.1 General method for the preparation of phenylthio-benzoic acid derivatives 8-9

To a stirred mixture of iodobenzoic acid **7** (4.00 mmol) and the appropriate thiophenol (8.00 mmol) in water (30 mL), KOH was added (12.00 mmol), followed by Cu (0.31 mmol). The reaction mixture was then refluxed for 24 h, cooled, filtered over celite and the filtrate acidified using concentrated HCl. The precipitate thus formed was collected by filtration, washed with water and purified as described below.

4-((4-Hydroxyphenyl)thio)benzoic acid (8)[1]

This compound was previously reported. Spectral data agree with those specified in literature. Purified by trituration in hot *n*-hexane. Yield: 95%, white solid. **^{1}H-NMR (DMSO-*d*$_6$)**: δ 6.89 (d, *J*= 8.6 Hz, 2H), 7.08 (d, *J*= 8.6 Hz, 2H), 7.39 (d, *J*= 8.7 Hz, 2H), 7.80 (d, *J*= 8.7 Hz, 2H), 10.08 (bs, 1H, Ph-O**H**), 12.92 (bs, 1H, Ph-COO**H**).

4-((3-(Trifluoromethyl)phenyl)thio)benzoic acid (9)

Purified by trituration in hot *n*-hexane. Yield: 89%, white solid. **^{1}H-NMR (DMSO-*d*$_6$)**: δ 7.40 (d, *J*= 8.3 Hz, 2H), 7.65-7.78 (m, 4H), 7.92 (d, *J*= 8.3 Hz, 2H), 13.04 (bs, 1H, Ph-COO**H**). **^{19}F-NMR** (**DMSO-*d*$_6$**): δ -61.31 (s, 3F). **^{13}C-NMR** (**DMSO-*d*$_6$**): δ 166.6 (C=O), 140.3 (C), 135.8 (CH), 134.8 (C), 130.9 (CH), 130.4 (CH), 130.3 (C), 129.4 (CH), 127.8 (m, CH), 124.9 (m, CH), 122.5 (m, C). MS [ESI, m/z]: 297.0 $[M-H]^-$.

1.1.2 General method for the preparation of amide derivatives 11a-i, 12a,h

Thionyl chloride (0.146 ml, 2.02 mmol) was added dropwise to a solution of the different phenylthio-benzoic acids **8-9** (1.62 mmol) in anhydrous DMA (6 ml) at −15 to −10 °C under argon atmosphere. The reaction mixture was stirred for 1 h at room temperature, and a solution of the different anilines **10a-i** (1.62 mmol) in anhydrous DMA (2 ml) was added dropwise to the above reaction solution. The resulting mixture was stirred at room temperature overnight. After completion of the reaction was established by TLC, the mixture was evaporated in vacuo, diluted with saturated aqueous $NaHCO_3$ (40 ml), and extracted with Et_2O (3×50 ml). The organic layers were combined and washed with 2M aqueous HCl (2x30 mL). The organic layer was dried over Na_2SO_4 and the solvent vas removed at reduced pressure. The crude residue was purified by flash column chromatography followed by recrystallization if required.

N-(4-Cyano-3-(trifluoromethyl)phenyl)-4-((4-hydroxyphenyl)thio)benzamide (11a). Purified by flash column chromatography eluting with n-hexane-EtOAc 100:0 v/v increasing to n-hexane-EtOAc 70:30 v/v followed by recrystallization from DCM. Yield: 30%, white solid. **^{1}H-NMR (DMSO-d_6)**: δ 6.90 (d, J= 8.7 Hz, 2H), 7.17 (d, J= 8.6 Hz, 2H), 7.40 (d, J= 8.7 Hz, 2H), 7.88 (d, J= 8.6 Hz, 2H), 8.13 (d, J= 8.6 Hz, 1H), 8.25 (dd, J= 8.6 Hz, 2Hz, 1H), 8.44 (d, J= 2Hz, 1H), 10.00 (bs, 1H), 10.82 (bs, 1H). **^{19}F-NMR (DMSO-d_6)**: δ -61.16 (s, 3F). **^{13}C-NMR (DMSO-d_6)**: δ 165.7 (C=O), 158.9 (C), 145.1 (C), 143.8 (C), 136.8 (CH), 136.3 (CH), 131.4 (m, C), 130.2 (C), 128.6 (CH), 125.4 (CH), 123.5 (m, C), 122.7 (m, CH), 118.2 (C), 117.4 (m, CH), 117.0 (CH), 115.7 (C), 101.7 (C). MS [ESI, m/z]: 415.1 $[M+H]^+$, 437.1 $[M+Na]^+$. HPLC (method 2): retention time = 27.32 min.

4-((4-Hydroxyphenyl)thio)-N-(4-nitro-3-(trifluoromethyl)phenyl)benzamide (11b). Purified by flash column chromatography eluting with n-hexane-EtOAc 100:0 v/v increasing to n-hexane-EtOAc 70:30 v/v. Yield: 79%, pale yellow solid. **^{1}H-NMR (DMSO-d_6)**: δ 6.85 (d, J= 8.6 Hz, 2H), 7.18 (d, J= 8.5 Hz, 2H), 7.41 (d, J= 8.6 Hz, 2H), 7.89 (d, J= 8.5 Hz, 2H), 8.24 (d, J= 9.0 Hz, 1H), 8.31 (d, J_1= 9.0 Hz, J_2= 2.1 Hz, 1H), 8.46 (d, J= 2.1 Hz, 1H), 10.01 (bs, 1H), 10.87 (bs, 1H). **^{19}F-NMR (DMSO-d_6)**: δ -58.64 (s, 3F). **^{13}C-NMR (DMSO-d_6)**: δ 165.6 (C=O), 158.7 (C), 145.2 (C), 143.8 (C), 141.4 (C), 136.8 (CH), 130.0 (C), 128.4 (CH), 127.5 (CH), 125.3 (CH), 123.0 (CH), 122.6 (m, C), 121.0 (m, C), 118.2 (CH), 118.0 (C), 116.8 (CH). MS [ESI, m/z]: 457.1 $[M+Na]^+$. HPLC (method 1): retention time = 23.23 min.

N-(3,5-bis(Trifluoromethyl)phenyl)-4-((4-hydroxyphenyl)thio)benzamide (11c). Purified by flash column chromatography eluting with n-hexane-EtOAc 100:0 v/v increasing to n-hexane-EtOAc 80:20 v/v. Yield: 78%, white solid. **^{1}H-NMR (DMSO-d_6)**: δ 6.91 (d, J= 8.6 Hz, 2H), 7.18 (d, J= 8.5 Hz, 2H), 7.41 (d, J= 8.6 Hz, 2H), 7.80 (s, 1H), 7.90 (d, J= 8.5 Hz, 2H), 8.50 (s, 2H), 10.02 (bs, 1H), 10.74 (bs, 1H). **^{19}F-NMR (DMSO-d_6)**: δ -61.60 (s, 6F). **^{13}C-NMR (DMSO-d_6)**: δ 165.6 (C=O), 158.9 (C), 144.9 (C), 141.1 (C), 136.8 (CH), 130.4 (m, C), 128.5 (CH), 125.5 (CH), 123.5 (m, C), 119.8 (CH), 118.3 (C), 117.1 (CH), 116.3 (CH). MS [ESI, m/z]: 458.1 $[M+H]^+$, 480.1 $[M+Na]^+$. HPLC (method 1): retention time = 26.26 min.

N-(2,5-bis-(Trifluoromethyl)phenyl)4-((4-hydroxyphenyl)thio)benzamide (11d).

Purified by flash column chromatography eluting with *n*-hexane-EtOAc 100:0 v/v increasing to *n*-hexane-EtOAc 80:20 v/v followed by recrystallization from MeOH/H_2O. Yield: 30%, white solid. **^{1}H-NMR (DMSO-*d*$_6$)**: δ 6.90 (d, *J*= 7.8 Hz, 2H), 7.17 (d, *J*= 7.8 Hz, 2H), 7.40 (d, *J*= 7.9 Hz, 2H), 7.85 (d, *J*= 7.9 Hz, 2H), 7.96 (d, *J*= 8.1 Hz, 1H), 7.98 (s, 1H), 8.05(d, *J*= 8.1 Hz, 1H), 10.0 (bs, 1H), 10.15 (bs, 1H). **^{19}F-NMR** (**DMSO-*d*$_6$**): δ -61.78 (s, 3F), -59.94 (s, 3F). **^{13}C-NMR** (**DMSO-*d*$_6$**): δ 165.9 (C=O), 158.8 (C), 144.5 (C), 137.0 (C), 136.7 (CH), 133.2 (m, C), 130.1 (C), 129.6 (m, C), 128.4 (CH), 128.2 (m, CH), 128.1 (CH), 127.7 (m, C), 126.3 (CH), 125.6 (CH), 124.1 (m, C), 118.5 (C), 117.0 (CH). MS [ESI, m/z]: 458.1 $[M+H]^+$. HPLC (method 1): retention time = 23.28 min.

4-((4-Hydroxyphenyl)thio)-*N*-(4-(pentafluorothio)phenyl)benzamide (11e).

Purified by flash column chromatography eluting with *n*-hexane-EtOAc 100:0 v/v increasing to *n*-hexane-EtOAc 80:20 v/v. Yield: 62%, white solid. **^{1}H-NMR (DMSO-*d*$_6$)**: δ 6.90 (d, *J*= 8.7 Hz, 2H), 7.16 (d, *J*= 8.6 Hz, 2H), 7.40 (d, *J*= 8.7 Hz, 2H), 7.85-7.90 (m, 2 collapsed d, 4H), 7.98 (d, *J*= 9.2 Hz, 2H), 9.99 (bs, 1H), 10.54 (bs, 1H). **^{19}F-NMR** (**DMSO-*d*$_6$**): δ 64.89 (d, *J*= 150.4 Hz, 4F), 88.69 (q, *J*= 150.4 Hz, 1F). **^{13}C-NMR** (**DMSO-*d*$_6$**): δ 164.4 (C=O), 158.8 (C), 147.4 (m, C), 144.5 (C), 142.4 (C), 136.7 (CH), 130.8 (C), 128.5 (CH), 126.5 (CH), 125.5 (CH), 119.7 (CH), 118.4 (C), 117.0 (CH). MS [ESI, m/z]: 448.1 $[M+H]^+$, 470.0 $[M+Na]^+$. HPLC (method 2): retention time = 28.26 min.

***N*-(3-(Pentafluorosulfanyl)phenyl)-4-((4-hydroxyphenyl)thio)benzamide (11f).**

Purified by flash column chromatography eluting with *n*-hexane-EtOAc 100:0 v/v increasing to *n*-hexane-EtOAc 70:30 v/v. Yield: 69%, white solid. **^{1}H-NMR (DMSO-*d*$_6$)**: δ 6.91 (d, *J*= 8.6 Hz, 2H), 7.17 (d, *J*= 8.5 Hz, 2H), 7.41 (d, *J*= 8.6 Hz, 2H), 7.56-7.64 (m, 2H), 7.88 (d, *J*= 8.5 Hz, 2H), 8.04 (d, *J*= 6.5 Hz, 1H), 8.42 (s, 2H), 9.99 (bs, 1H), 10.51 (bs, 1H). **^{19}F-NMR** (**DMSO-*d*$_6$**): δ 63.78 (d, *J*= 150.5 Hz, 4F), 88.57 (q, *J*= 150.5 Hz, 1F). **^{13}C-NMR** (**DMSO-*d*$_6$**): δ 165.3 (C=O), 158.9 (C), 152.9 (m, C), 144.5 (C), 139.8 (C), 136.8 (CH), 130.8 (C), 129.6 (CH), 128.5 (CH), 125.5 (CH), 123.6 (CH), 120.5 (CH), 118.5 (C), 117.0 (CH). MS [ESI, m/z]: 448.0 $[M+H]^+$, 470.0 $[M+Na]^+$. HPLC (method 1): retention time = 23.29 min.

4-((4-Hydroxyphenyl)thio)-*N*-(4-(trifluoromethyl)phenyl)benzamide (11g).

Purified by flash column chromatography eluting with *n*-hexane-EtOAc 100:0 v/v increasing to *n*-hexane-EtOAc 70:30 v/v. Yield: 43%, white solid. **^{1}H-NMR (DMSO-*d*$_6$)**: δ 6.90 (d, *J*=

8.7 Hz, 2H), 7.16 (d, *J*= 8.6 Hz, 2H), 7.40 (d, *J*= 8.7 Hz, 2H), 7.71 (d, *J*= 8.6 Hz, 2H), 7.87 (d, *J*= 8.6 Hz, 2H), 7.98 (d, *J*= 8.6 Hz, 2H), 9.98 (bs, 1H), 10.47 (bs, 1H). **^{19}F-NMR** (**DMSO-*d*$_6$**): δ -60.32 (s, 3F). **^{13}C-NMR** (**DMSO-*d*$_6$**): δ 165.3 (C=O), 158.8 (C), 144.3 (C), 142.7 (C), 136.7 (CH), 131.0 (C), 128.5 (CH), 127.6 (m, CH), 125.8 (m, C), 125.5 (C), 120.0 (CH), 118.5 (C), 117.0 (CH). MS [ESI, m/z]: 390.1 $[M+H]^+$, 412.1 $[M+Na]^+$. HPLC (method 2): retention time = 27.34 min.

4-((4-Hydroxyphenyl)thio)-*N*-(3-(trifluoromethyl)phenyl)benzamide (11h).

Purified by flash column chromatography eluting with *n*-hexane-EtOAc 100:0 v/v increasing to *n*-hexane-EtOAc 70:30 v/v. Yield: 71%, pale yellow solid. **^{1}H-NMR (DMSO-*d*$_6$)**: δ 6.92 (d, *J*= 8.5 Hz, 2H), 7.17 (d, *J*= 8.2 Hz, 2H), 7.40 (d, *J*= 8.5 Hz, 2H), 7.49-7.52 (m, 1H), 7.59 (apparent t, *J*= 8.0 Hz, 1H), 7.87 (d, *J*= 8.2 Hz, 2H), 8.00-8.04 (m, 1H), 8.23 (s, 1H), 9.99 (bs, 1H), 10.45 (bs, 1H). **^{19}F-NMR** (**DMSO-*d*$_6$**): δ -61.33 (s, 3F). **^{13}C-NMR** (**DMSO-*d*$_6$**): δ 165.3 (C=O), 158.8 (C), 144.3 (C), 139.9 (C), 136.7 (CH), 131.0 (C), 129.8 (CH), 129.32 (m, C), 128.44 (CH), 125.5 (C), 123.7 (CH), 119.84 (m, CH), 118.5 (C), 117.0 (CH), 116.26 (m, CH). MS [ESI, m/z]: 390.1 $[M+H]^+$, 412.1 $[M+Na]^+$. HPLC (method 1): retention time = 22.43 min.

4-((4-Hydroxyphenyl)thio)-*N*-(2-(trifluoromethyl)phenyl)benzamide (11i).

Purified by flash column chromatography eluting with *n*-hexane-EtOAc 100:0 v/v increasing to *n*-hexane-EtOAc 70:30 v/v. Yield: 47%, white solid. **^{1}H-NMR (DMSO-*d*$_6$)**: δ 6.89 (d, *J*= 8.5 Hz, 2H), 7.18 (d, *J*= 8.7 Hz, 2H), 7.43 (d, *J*= 8.5 Hz, 2H), 7.51-7.58 (m, 2H), 7.70-7.73 (m, 1H), 7.81 (d, *J*= 7.8 Hz, 1H), 7.86 (d, *J*= 8.7 Hz, 2H), 10.02 (bs, 1H), 10.07 (bs, 1H). **^{19}F-NMR** (**DMSO-*d*$_6$**): δ -59.33 (s, 3F). **^{13}C-NMR** (**DMSO-*d*$_6$**): δ 165.6 (C=O), 158.8 (C), 144.1 (C), 136.6 (CH), 135.7 (C), 133.0 (CH), 131.1 (CH), 130.5 (C), 128.3 (CH), 127.3 (CH), 126.4 (m, CH), 126.3 (m, C), 125.6 (CH), 118.6 (C), 117.0 (CH). MS [ESI, m/z]: 390.1 $[M+H]^+$, 412.1 $[M+Na]^+$. HPLC (method 1): retention time = 20.95 min.

***N*-(4-Cyano-3-(trifluoromethyl)phenyl)-4-((3-trifluoromethyl)phenyl)thio)benzamide (12a).**

Purified by flash column chromatography eluting with *n*-hexane-EtOAc 100:0 v/v increasing to *n*-hexane-EtOAc 80:20 v/v. Yield: 62%, white solid. **^{1}H-NMR (DMSO-*d*$_6$)**: δ 7.50 (d, *J*= 8.5 Hz, 2H), 7.66-7.79 (m, 4H), 7.99 (d, *J*= 8.5 Hz, 2H), 8.15 (d, *J*= 8.8 Hz, 1H), 8.27 (dd, *J*= 8.8 Hz, 1.5 Hz, 1H), 8.45 (d, *J*= 1.5 Hz, 1H), 10.94 (bs, 1H, NH). **^{19}F-NMR** (**DMSO-*d*$_6$**): δ -

61.16 (s, 3F), -61.31 (s, 3F). **^{13}C-NMR** (**DMSO-*d*$_6$**): δ 165.6 (C=O), 143.7 (C), 139.6 (C), 136.4 (CH), 135.5 (CH), 135.1 (C), 132.5 (C), 131.5 (m, C), 130.9 (CH), 130.3 (m, C), 129.7 (CH), 129.1 (CH), 127.5 (m, CH), 125.7 (m, C), 124.8 (m, CH), 123.5 (m, C), 122.8 (CH), 117.5 (m, CH), 115.7 (C), 101.9 (C). MS [ESI, m/z]: 467.1 $[M+H]^+$, 489.0 $[M+Na]^+$. HPLC (method 1): retention time = 26.77 min.

***N*-(3-(Trifluoromethyl)phenyl)-4-((3-(trifluoromethyl)phenyl)thio)benzamide (12h).** Purified by flash column chromatography eluting with *n*-hexane-EtOAc 100:0 v/v increasing to *n*-hexane-EtOAc 90:10 v/v. Yield: 72%, white solid. **^{1}H-NMR (DMSO-*d*$_6$)**: δ 7.46-7.49 (m, 1H), 7.51 (d, *J*= 8.5 Hz, 2H), 7.58-7.62 (m, 1H), 7.65-7.86 (m, 4H), 7.17 (d, *J*= 8.5 Hz, 2H), 8.02 (d, *J*= 8.5 Hz, 2H), 8.03-8.06 (m, 1H), 8.25 (s, 1H), 10.58 (bs, 1H, NH). **^{19}F-NMR** (**DMSO-*d*$_6$**): δ -63.32 (s, 3F), -63.36 (s, 3F). **^{13}C-NMR** (**DMSO-*d*$_6$**): δ 165.0 (C=O), 148.8 (C), 147.4 (C), 142.6 (C), 141.9 (C), 139.6 (m, C) 137.2 (C), 131.8 (CH), 131.9 (CH), 130.9 (m, CH), 130.5 (m, C), 129.9 (m, CH), 129.2 (CH), 129.0 (CH), 128.2 (CH), 125.1 (m, C), 123.9 (m, CH), 120.3 (m, CH), 116.3 (m, CH). MS [ESI, m/z]: 442.1 $[M+H]^+$, 464.1 $[M+Na]^+$. HPLC (method 1): retention time = 27.04 min.

1.1.3 General method for the preparation of sulfones derivatives 13a-i, 14e,i and 15a,h.

To a stirring solution of the different sulfide **11a-i**, **12a**, **h** (0.24 mmol) in DCM (3 mL) was added portionwise mCPBA (< 77%) (0.51 mmol), maintaining the temperature at 25 °C. The solution was stirred overnight. The mixture was evaporated *in vacuo*, diluted with saturated $NaHCO_3$ solution (20 ml), and extracted with Et_2O (3×50 ml). The combined organic layers were dried over Na_2SO_4, and finally concentrated *in vacuo*. The crude residue was purified by flash column chromatography, precipitation or recrystallization. In two cases, compounds **11e** and **11i**, the product presenting the partial oxidation of sulphur was isolated.

***N*-(4-Cyano-3-(trifluoromethyl)phenyl)-4-((4-hydroxyphenyl)sulfonyl benzene (13a).** Purified by flash column chromatography eluting with *n*-hexane-EtOAc 100:0 v/v increasing to *n*-hexane-EtOAc 50:50 v/v. Yield: 38%, white solid. **^{1}H-NMR (DMSO-*d*$_6$)**: δ 6.95 (d, *J*= 8.8 Hz, 2H), 7.83 (d, *J*= 8.8 Hz, 2H), 8.08 (d, *J*= 8.5 Hz, 2H), 8.13 (d, *J*= 8.5 Hz, 2H), 8.16 (d, *J*= 8.7 Hz, 1H), 8.24 (dd, *J*= 8.7 Hz, 1.8 Hz, 1H), 8.42 (d, *J*= 1.8 Hz, 1H), 10.71 (bs, 1H), 11.08 (bs, 1H). **^{19}F-NMR** (**DMSO-*d*$_6$**): δ -61.18 (s, 3F). **^{13}C-NMR** (**DMSO-*d*$_6$**): δ 165.2 (C=O),

162.4 (C), 145.2 (C), 143.4 (C), 137.9 (C), 136.4 (CH), 131.4 (m, C), 130.1 (CH), 129.9 (C), 129.1 (CH), 127.1 (CH), 122.9 (CH), 122.0 (m, C), 117.5 (m, CH), 116.2 (CH), 115.6 (C), 102.3 (C). MS [ESI, m/z]: 447.1 $[M+H]^+$, 469.0 $[M+Na]^+$. HPLC (method 2): retention time = 23.90 min.

4-((4-Hydroxyphenyl)sulfonyl)-*N*-(4-nitro-3-(trifluoromethyl)phenyl)benzamide (13b). Purified by flash column chromatography eluting with *n*-hexane-EtOAc 100:0 v/v increasing to *n*-hexane-EtOAc 60:40 v/v. Yield: 53%, white solid. **^{1}H-NMR (DMSO-*d*$_6$)**: δ 6.96 (d, *J*= 8.8 Hz, 2H), 7.83 (d, *J*= 8.8 Hz, 2H), 8.09 (d, *J*= 8.4 Hz, 2H), 8.15 (d, *J*= 8.4 Hz, 2H), 8.25-8.31 (m, 2H), 8.44 (d, *J*= 1.6 Hz, 1H), 10.73 (bs, 1H), 11.10 (bs, 1H). **^{19}F-NMR** (**DMSO-*d*$_6$**): δ -59.06 (s, 3F). **^{13}C-NMR** (**DMSO-*d*$_6$**): δ 165.2 (C=O), 162.4 (C), 145.2 (C), 143.4 (C), 141.8 (C), 137.8 (C), 130.1 (CH), 129.9 (C), 129.1 (CH), 127.5 (CH), 127.1 (CH), 123.4 (CH), 122.9 (m, C), 120.7 (m, C), 118.4 (m, CH), 116.2 (CH). MS [ESI, m/z]: 467.1 $[M+H]^+$, 489.1 $[M+Na]^+$. HPLC (method 1): retention time = 20.24 min.

***N*-(3,5-bis(Trifluoromethyl)phenyl)-4-((4-hydroxyphenyl)sulfonyl)benzamide (13c).** Purified by recrystallization from DCM. Yield: 48%, white solid. **^{1}H-NMR (DMSO-*d*$_6$)**: δ 6.96 (d, *J*= 8.7 Hz, 2H), 7.80-7.86 (m, 3H), 8.07 (d, *J*= 8.4 Hz, 2H), 8.17 (d, *J*= 8.4 Hz, 2H), 8.48 (s, 2H), 10.74 (bs, 1H), 10.99 (bs, 1H). **^{19}F-NMR** (**DMSO-*d*$_6$**): δ -61.70 (s, 6F). **^{13}C-NMR** (**DMSO-*d*$_6$**): δ 165.0 (C=O), 162.5 (C), 145.2 (C), 140.7 (C), 137.9 (C), 130.8 (m, C), 130.1 (CH), 129.9 (C), 129.0 (CH), 127.1 (CH), 123.56 (m, C), 119.9 (CH), 116.8 (CH), 116.3 (CH). MS [ESI, m/z]: 490.0 $[M+H]^+$, 512.0 $[M+Na]^+$. HPLC (method 1): retention time = 22.05 min.

***N*-(2,5-bis-(Trifluoromethyl)phenyl)4-((4-hydroxyphenyl)sulfonyl)benzamide (13d).** Purified by flash column chromatography eluting with *n*-hexane-EtOAc 100:0 v/v increasing to *n*-hexane-EtOAc 60:40 v/v followed by recrystallization from MeOH/H_2O. Yield: 15%, white solid. **^{1}H-NMR (DMSO-*d*$_6$)**: δ 6.96 (d, *J*= 8.9 Hz, 2H), 7.82 (d, *J*= 8.9 Hz, 2H), 7.95 (d, *J*= 8.3 Hz, 2H), 8.03 (s, 1H), 8.05-8.12 (m, 5H), 10.62 (bs, 1H), 10.67 (bs, 1H). **^{19}F-NMR** (**DMSO-*d*$_6$**): δ -61.77 (s, 3F), -60.01 (s, 3F). **^{13}C-NMR** (**DMSO-*d*$_6$**): δ 165.5 (C=O), 162.3 (C), 145.0 (C), 137.6 (C), 136.5 (C), 133.2 (m, C), 130.1 (CH), 130.0 (C), 129.8 (m, C), 128.9 (CH), 128.2 (m, CH), 127.9 (m, CH), 127.1 (CH), 124.5 (m, CH), 124.1 (m, C), 121.7 (m, C), 116.2 (CH). MS [ESI, m/z]: 490.1 $[M+H]^+$, 512.0 $[M+Na]^+$. HPLC (method 1): retention time = 19.36 min.

4-((4-Hydroxyphenyl)sulfonyl)-*N*-(4-(pentafluorothio)phenyl)benzamide (13e) and 4-((4-Hydroxyphenyl)sulfinyl)-*N*-(4-(pentafluorothio)phenyl)benzamide (14e).

Compound **13e** purified by flash column chromatography eluting with *n*-hexane-EtOAc 100:0 v/v increasing to *n*-hexane-EtOAc 50:50 v/v followed by precipitation from EtOAc-*n*-hexane. Yield: 22%, white solid. Compound **14e** purified by flash column chromatography eluting with *n*-hexane-EtOAc 100:0 v/v increasing to *n*-hexane-EtOAc 40:60 v/v. Yield: 13%, white solid.

Compound **13e**: **^{1}H-NMR (DMSO-d_6)**: δ 6.95 (d, *J*= 8.4 Hz, 2H), 7.82 (d, *J*= 8.4 Hz, 2H), 7.91 (d, *J*= 8.9 Hz, 2H), 7.98 (d, *J*= 8.9 Hz, 2H), 8.05 (d, *J*= 8.1 Hz, 2H), 8.11 (d, *J*= 8.1 Hz, 2H), 10.71 (bs, 1H), 10.81 (bs, 1H). **^{19}F-NMR** (**DMSO-d_6**): δ 64.80 (d, *J*= 150.5 Hz, 4F), 88.45 (q, *J*= 150.5 Hz, 1F). **^{13}C-NMR** (**DMSO-d_6**): δ 164.9 (C=O), 162.4(C), 147.7 (m, C), 144.9 (C), 142.0 (C), 138.4 (C), 130.1 (CH), 130.0 (C), 129.0 (CH), 127.0 (CH), 126.6 (m, CH), 119.9 (CH), 116.2 (CH). MS [ESI, m/z]: 480.0 $[M+H]^+$, 502.0 $[M+Na]^+$. HPLC (method 1): retention time = 20.35 min.

Compound **14e**: **^{1}H-NMR (DMSO-d_6)**: δ 6.90 (d, *J*= 8.5 Hz, 2H), 7.55 (d, *J*= 8.5 Hz, 2H), 7.80 (d, *J*= 8.3 Hz, 2H), 7.90 (d, *J*= 9.0 Hz, 2H), 7.98 (d, *J*= 9.0 Hz, 2H), 8.07 (d, *J*= 8.3 Hz, 2H), 10.22 (bs, 1H), 10.73 (bs, 1H). **^{19}F-NMR** (**DMSO-d_6**): δ 64.83 (d, *J*= 150.4 Hz, 4F), 88.52 (q, *J*= 150.4 Hz, 1F). **^{13}C-NMR** (**DMSO-d_6**): δ 165.3 (C=O), 160.4(C), 150.1 (C), 147.6 (m, C), 142.2 (C), 136.3 (C), 134.5 (C), 129.0 (m, CH), 128.7 (CH), 126.6 (CH), 123.8 (CH), 119.8 (CH), 116.3 (CH). MS [ESI, m/z]: 464.0 $[M+H]^+$, 486.0 $[M+Na]^+$. HPLC (method 1): retention time = 18.25 min.

***N*-(3-(Pentafluorosulfanyl)phenyl)-4-((4-hydroxyphenyl)sulfonyl)benzamide (13f).**

Purified by recrystallization from EtOAc/*n*-hexane. Yield: 67%, white solid. **^{1}H-NMR (DMSO-d_6)**: δ 6.99 (d, *J*= 8.6 Hz, 2H), 7.58-7.75 (m, 2H), 7.85 (d, *J*= 8.6 Hz, 2H), 8.00-8.10 (m, 3H), 8.14 (d, *J*= 8.5 Hz, 2H), 8.41 (apparent t, *J*= 1.9 Hz, 1H), 10.71 (bs, 1H), 10.75 (bs, 1H). **^{19}F-NMR** (**DMSO-d_6**): δ 63.50 (d, *J*= 150.7 Hz, 4F), 87.99 (q, *J*= 150.7 Hz, 1F). **^{13}C-NMR** (**DMSO-d_6**): δ 164.8 (C=O), 162.4 (C), 144.9 (C), 139.4 (C), 138.4 (C), 130.1 (CH), 130.0 (C), 129.8 (CH), 128.9 (CH), 127.0 (CH), 123.7 (CH), 120.9 (CH), 117.1 (CH), 116.3 (CH). MS [ESI, m/z]: 480.0 $[M+H]^+$, 502.0 $[M+Na]^+$. HPLC (method 1): retention time = 20.12 min.

4-((4-Hydroxyphenyl)sulfonyl)-*N*-(4-(trifluoromethyl)phenyl)benzamide (13g).

Purified by precipitation from EtOAc-*n*-hexane-. Yield: 43%, white solid. **^{1}H-NMR (DMSO-*d*$_6$)**: δ 6.95 (d, *J*= 8.8 Hz, 2H), 7.74 (d, *J*= 8.5 Hz, 2H), 7.84 (d, *J*= 8.8 Hz, 2H), 7.98 (d, *J*= 8.5 Hz, 2H), 8.05 (d, *J*= 8.5 Hz, 2H), 8.11 (d, *J*= 8.5 Hz, 2H), 10.47 (bs, 2H, Ph-O**H** and NH). **^{19}F-NMR** (**DMSO-*d*$_6$**): δ -60.32 (s, 3F). **^{13}C-NMR** (**DMSO-*d*$_6$**): δ 164.8 (C=O), 162.5 (C), 144.9 (C), 142.3 (C), 138.6 (C), 130.0 (CH), 129.9 (C), 129.0 (CH), 127.0 (CH), 125.9 (m, CH), 125.2 (m, C), 123.8 (m, C), 120.1 (CH), 116.2 (CH). MS [ESI, m/z]: 422.1 $[M+H]^+$, 444.1 $[M+Na]^+$. HPLC (method 2): retention time = 24.79 min.

4-((4-Hydroxyphenyl)sulfonyl)-*N*-(3-(trifluoromethyl)phenyl)benzamide (13h).

Purified by flash column chromatography eluting with *n*-hexane-EtOAc 100:0 v/v increasing to *n*-hexane-EtOAc 60:40 v/v. Yield: 57%, white solid. **^{1}H-NMR (DMSO-*d*$_6$)**: δ 6.99 (d, *J*= 8.4 Hz, 2H), 7.49 (d, *J*= 7.8 Hz, 1H), 7.62 (apparent t, *J*= 8.0 Hz, 1H), 7.85 (d, *J*= 8.4 Hz, 2H), 7.98-8.04 (m, 1H), 8.08 (d, *J*= 8.2 Hz, 2H), 8.13 (d, *J*= 8.2 Hz, 2H), 8.22 (s, 1H), 10.68 (bs, 1H), 10.73 (bs, 1H). **^{19}F-NMR** (**DMSO-*d*$_6$**): δ -61.36 (s, 3F). **^{13}C-NMR** (**DMSO-*d*$_6$**): δ 164.7 (C=O), 162.3 (C), 144.8 (C), 139.5 (C), 138.5 (C), 130.1 (CH), 129.9 (m, CH), 129.2 (m, C), 128.9 (CH), 127.0 (CH), 123.8 (CH), 120.3 (CH), 116.4 (CH), 116.3 (m, CH). MS [ESI, m/z]: 422.1 $[M+H]^+$, 444.0 $[M+Na]^+$. HPLC (method 1): retention time = 19.13 min.

4-((4-Hydroxyphenyl)sulfonyl)-*N*-(2-(trifluoromethyl)phenyl)benzamide (13i) and 4-((4-Hydroxyphenyl)sulfinyl)-*N*-(2-(trifluoromethyl)phenyl)benzamide (14i).

Compound **13i** purified by flash column chromatography eluting with *n*-hexane-EtOAc 100:0 v/v increasing to *n*-hexane-EtOAc 70:30 v/v. Yield: 39%, white solid. Compound **14i** purified by flash column chromatography eluting with *n*-hexane-EtOAc 100:0 v/v increasing to *n*-hexane-EtOAc 65:35 v/v. Yield: 27%, white waxy solid.

Compound **13i**: **^{1}H-NMR (DMSO-*d*$_6$)**: δ 6.96 (d, *J*= 8.7 Hz, 2H), 7.51-7.60 (m, 2H), 7.65-7.63 (m, 1H), 7.81-7.88 (m, 3H), 8.01-8.17 (m, 4H), 10.38 (bs, 1H), 10.79 (bs, 1H). **^{19}F-NMR** (**DMSO-*d*$_6$**): δ -59.39 (s, 3F). **^{13}C-NMR** (**DMSO-*d*$_6$**): δ 165.2 (C=O), 162.3 (C), 144.8 (C), 137.9 (C), 135.2 (C), 133.2 (CH), 131.1 (CH), 130.0 (CH), 128.8 (CH), 127.7 (CH), 126.5 (m, CH), 126.2 (m, C), 126.0 (C), 122.4 (C), 116.2 (CH). MS [ESI, m/z]: 422.1 $[M+H]^+$, 444.1 $[M+Na]^+$. HPLC (method 1): retention time = 16.55 min.

Compound **14i**: **^{1}H-NMR ($CDCl_3$)**: δ 6.95 (d, *J*= 8.6 Hz, 2H), 7.39-7.43 (m, 1H), 7.45 (d, *J*= 8.6 Hz, 2H), 5.56-7.63 (m, 1H), 7.67 (d, *J*= 7.7 Hz, 1H), 7.70 (d, *J*= 8.7 Hz, 2H), 7.95 (d, *J*=

8.7 Hz, 2H), 8.24 (d, *J*= 8.2 Hz, 1H), 8.31 (bs, 1H), 8.53 (bs, 1H). **^{19}F-NMR** (**$CDCl_3$**): δ -60.36 (s, 3F). **^{13}C-NMR** (**$CDCl_3$**): δ 164.9 (C=O), 160.7 (C), 149.1 (C), 136.3 (C), 134.7 (C), 133.6 (C), 133.0 (CH), 128.2 (CH), 127.9 (CH), 126.3 (m, CH), 126.2 (CH), 125.4 (CH), 125.1 (m, CH), 122.9 (C), 121.4 (m, C), 117.2 (CH). MS [ESI, m/z]: 406.1 $[M+H]^+$, 428.1 $[M+Na]^+$. HPLC (method 1): retention time = 13.93 min.

***N*-(4-Cyano-3-(trifluoromethyl)phenyl)-4-((3-trifluoromethyl)phenyl)sulfonyl) benzamide (15a).**

Purified by flash column chromatography eluting with *n*-hexane-EtOAc 100:0 v/v increasing to *n*-hexane-EtOAc 70:30 v/v. Yield: 48%, white solid. **^{1}H-NMR (DMSO-*d*$_6$)**: δ 7.90-7.92 (m, collapsed dd, 1H), 8.11-8.19 (m, 4H), 8.23 (dd, *J*= 8.5 Hz, 1.6 Hz, 1H), 8.27 (d, *J*= 8.4 Hz, 2H), 8.32-8.37 (m, 2H), 8.41 (d, *J*= 1.6 Hz, 1H), 11.11 (bs, 1H, NH). **^{19}F-NMR** (**DMSO-*d*$_6$**): δ -61.20 (s, 3F), -61.31 (s, 3F). **^{13}C-NMR** (**DMSO-*d*$_6$**): δ 165.1 (C=O), 143.4 (C), 143.0 (C), 141.8 (C), 138.9 (C), 136.4 (CH), 131.8 (CH), 131.5 (CH), 131.4 (m, C), 130.8 (m, CH), 130.3 (m, C), 129.4 (CH), 128.0 (CH), 124.2 (m, C), 124.0 (m, CH), 123.5 (m, C), 123.0 (CH), 117.5 (m, CH), 115.6 (C), 102.3(C). MS [ESI, m/z]: 499.0 $[M+H]^+$, 521.0 $[M+Na]^+$. HPLC (method 1): retention time = 23.00 min.

***N*-(3-(Trifluoromethyl)phenyl)-4-((3-(trifluoromethyl)phenyl)sulfonyl)benzamide (15h).**

Purified by flash column chromatography eluting with *n*-hexane-EtOAc 100:0 v/v increasing to *n*-hexane-EtOAc 70:30 v/v. Yield: 75%, white solid. **^{1}H-NMR (DMSO-*d*$_6$)**: δ 7.49 (d, *J*= 7.8 Hz, 1H), 7.62 (apparent t, *J*= 8.0 Hz, 1H), 7.92 (apparent t, *J*= 7.9 Hz, 1H), 8.01 (d, *J*= 8.0 Hz, 1H), 8.13-8.19 (m, 3H), 8.22 (s, 1H), 8.26 (d, *J*= 8.7 Hz, 2H), 8.31-8.38 (m, 2H), 10.75 (bs, 1H). **^{19}F-NMR** (**DMSO-*d*$_6$**): δ -61.25 (s, 3F), -61.33 (s, 3F). **^{13}C-NMR** (**DMSO-*d*$_6$**): δ 164.5 (C=O), 142.6 (C), 141.9 (C), 139.5 (C), 139.4 (C), 131.8 (CH), 131.5 (CH), 130.8 (m, CH), 130.4 (m, C), 129.9 (CH), 129.5 (C), 129.2 (CH), 127.9 (CH), 124.0 (m, C), 123.9 (CH), 122.9 (C), 122.0 (m, CH), 120.3 (m, CH), 116.3 (m, CH). MS [ESI, m/z]: 474.1 $[M+H]^+$, 496.1 $[M+Na]^+$. HPLC (method 1): retention time = 23.31 min.

1.2 Molecular Modelling

All molecular docking studies were performed on a Viglen Genie Intel®Core™ i7-3770 vPro CPU@ 3.40 GHz x 8 running Ubuntu 14.04. The AR structure in its close conformation was

downloaded from the PDB data bank (http://www.rcsb.org/; PDB code 1Z95). Hydrogen atoms were added to the protein, using the Protonate 3D routine of the Molecular Operating Environment (MOE 2015.10).[2] Preparation and validation of the homology model of the open AR conformation has been previously published by our group.[3] Ligand structures were built with MOE2015.10 and minimized using the MMFF94x force field until a RMSD gradient of 0.05 kcal mol−1 Å−1 was reached. Docking studies, in both homology model and crystal structure, were performed using Plants (Protein-Ligand ANT System), an open source docking program.[4] The binding site center was defined from the co-crystallized bicalutamide PDB coordinates (PDB ID: IZ95), taking as center the spatial coordinates of the chiral carbon (29.681, 0.203, 4.612) and then the radius was extended to 12 Å, in order to include in the docking site all the important amino acids. A mol2 format for the Plants algorithm of our homology model and for the crystal structure of the AR closed conformation were prepared using MOE2105.10. A ligand database in mol2 format was used as input for the docking calculations. Ligand docking was performed using the default values, and no water molecules were considered. Five output solutions were kept for each compound, and visual inspection in MOE was used to identify the interaction between ligand and protein. The flexible alignment studies were performed using MOE 2015.10. The MOE flexible alignment tool generates different possible conformations for a test compound that could overlap with the assigned template (active binding pose of bicalutamide and enzalutamide in our case). The quality of the alignment is evaluated by a score which is a sum of the internal strain of the obtained conformation (the more negative, the better) and the overlap of molecular features (aromatic regions, donors/acceptors). MOE, for each alignment performed, evaluates the average internal energy of the ligands U, the similarity score F (the more negative this value, the better two structures overlap) and the value S (sum of U and F values obtained for each alignment). A good alignment should present a dU value (the average strain energy of the molecules in the alignment in kcal/mol) lower than 1 kcal/mol, meaning that the obtained conformation is energetically favoured. In our case, we kept our template rigid (bicalutamide and enzalutamide) and the flexible alignment of compound **13a** was run. The scoring results obtained for **compound 13a** are reported in the table below.

Template	U (kcal/mol)	F	S	dU
Bicalutamide	41.4829	-136.9531	-95.4702	0.0
Enzalutamide	41.4829	-149.6317	-108.1487	0.0

Table S1. Scoring results obtained for **compound 13a**

1.3 Antiproliferative assay

All the antiproliferative assays were performed by **Oncotest GmbH**, according to their internal procedures.[5]

Compound Handling: Stock solutions for all inhibitors were prepared in DMSO at a concentration of 33 mM and aliquots of 200 µl were finally stored at -20°C. All compounds were well soluble at this concentration in DMSO and precipitation was not observed for any compound dissolved in 100% DMSO. Aliquots of the stock solution were thawed on the day of use and stored at room temperature prior to and during treatment. The subsequent dilutions were done with complete RPMI1640 cell culture medium. The DMSO stock solution was first diluted 1:22 (corresponding to 4.5% v/v DMSO). Starting with this solution, serial dilutions in half-log steps with cell culture medium were done using an intermediate dilution plate. Finally, 10 µl taken from the intermediate dilution plate were transferred to 140 µl / well of the cell culture plate. Thus, at the highest test concentration the DMSO stock was diluted 1:330, corresponding to a maximum DMSO concentration of 0.3 % v/v in the assay. Solubility of the diluted compounds in the assay was monitored under the microscope by visual inspection of the treated cell lines after the 4 days incubation. Precipitation as observed by crystalline structures was noticed at concentrations of 30 µM and of 100 µM for some compounds. At lower concentrations no precipitation was seen for any compound.

Tumor Cell lines: The cell line panel used for assessing the novel inhibitors comprised four cell lines derived from human prostate cancers. Details on the characteristics of the tumor cell lines used in the present study are shown below.

Tumor Designation	Tumor Number	Histology	Primary/ Metastasis/ Recurrent	Androgen Sensitivity	Differentiation	Androgen receptor expression	Gender
PRXF	DU-145	prostate carcinoma	not known	insensitive	poorly differentiated	no	male
PRXF	22Rv1	prostate carcinoma	not known	partially sensitive	not known	yes	male
PRXF	LNCaP	prostate carcinoma	not known	sensitive	poorly differentiated	yes	male
PRXF	VCaP	prostate carcinoma	Metastasis	sensitive	not known	?	male

Authenticity of cell lines was proven at the DSMZ by STR (short tandem repeat) analysis, a PCR based DNA-fingerprinting methodology. [6-7] Cell lines were routinely passaged once or twice weekly and maintained in culture for up to 20 passages. All cells were grown at 37°C in a humidified atmosphere with 5% CO_2 in RPMI 1640 medium (25 mM HEPES, with L-glutamine, #FG1385, Biochrom, Berlin, Germany) supplemented with 10% (v/v) fetal calf serum (Sigma, Taufkirchen, Germany) and 0.1 mg/mL gentamicin (Life Technologies, Karlsruhe, Germany).

Cell Proliferation Assay: A modified propidium iodide (PI) based monolayer assay was used to assess the anti-cancer activity of the compounds.[8] Briefly, cells were harvested from exponential phase cultures, counted and plated in 96-well flat-bottom micro titer plates at a cell density of 4.000 – 30.000 cells/well. After a 24 h recovery period to allow the cells to resume exponential growth, 10 µl of culture medium (six control wells/plate) or culture medium with test compound were added. The compounds were applied in half-log increments at 10 concentrations in triplicate. After a total treatment period of 96 h, cells were washed with 200 µl PBS to remove dead cells and debris. Then, 200 µl of a solution containing 7 µg/ml propidium iodide (PI) and 0.1% (v/v) Triton X-100 was added. After an incubation period of 1-2 hours at room temperature, fluorescence (FU) was measured using the EnSpire Multimode Plate Reader (excitation λ= 530 nm, emission λ= 620 nm) to quantify the amount of attached viable cells. IC_{50} values were calculated by 4 parameter non-linear curve fit using Oncotest Warehouse Software. For calculation of mean IC_{50} values the geometric mean was used.

Data Evaluation: An assay was considered fully evaluable if the following quality control criteria were fulfilled:

- Z'-factor calculated within the assay plate ≥0.5[9]
- fluorescence intensity of >500 U from the untreated control wells, equivalent to a control/background ratio >3.0
- coefficient of variation in the growth control wells ≤30

Compound effects were expressed in terms of the percentage of the fluorescence signal, obtained by comparison of the mean signal in the treated wells with the mean signal of the untreated controls (expressed by the test-versus-control value, T/C-value [%]):

$$\frac{T}{C}[\%] = \frac{\text{mean fluorescence signal}_{\text{treated group}}}{\text{mean fluorescence signal}_{\text{control group}}} \cdot 100$$

IC values are reported as absolute IC_{50} values. The absolute IC_{50} value reflects the concentration of the test compound that achieves T/C=50%. The calculation was done by a 4

parameter non-linear curve fit (Oncotest Data Warehouse Software). The overall potency of a compound was determined by the geometric mean IC_{50} value of all individual IC_{50} values. If an IC_{50} value could not be determined within the examined dose range (because a compound was either too active or lacked activity), the lowest or highest concentration studied was used for calculation of the geometric mean value.

1.4 Cytotoxicity assay

Cytotoxicity was evaluated using a Cell Titer Blue Viability Assay**.** The Cell Titer-Blue Cell Viability Assay represents a fluorometric method for measuring the number of viable cells. The assay is based on the ability of viable cells to reduce resazurin into resorufin, which is fluorescent. Cells which are not viable rapidly lose metabolic capacity and ability to convert resazurin, and thus do not generate a fluorescent signal.

The human embryonic kidney cells HEK293 were cultured in Dulbecco-s Modified Eagle Medium (Gibco®) enriched with 10% v/v Fetal Bovine Serum (FBS, Sigma Dorset UK) and L-Glutamine (2 mM, Invitrogen) in 25 cm^2 cell culture flask (T25) (Nunc, Thermo). Adherent cells were removed from culture plate when they had reached 80-100% confluence using 2 ml of 0.05% Trypsin/EDTA. Cells were incubated for around 5 minutes at 37°C. When all cells were detached, at least 4 volumes of complete growth medium were added to inactivate the trypsin. Cells were transferred to a 15 ml falcon tube (Nunc) and counted using a Fastread 10-chamber counting grid (Immune Systems). The average of four counting squares was taken and multiplied by the required factor (10,000) to obtain the number of cells per ml. Cells were plated in 96 well plates at a concentration of 100,000 cells/ml to achieve an appropriate confluence for assay the next day. After 24 h incubation, cells were treated with controls and drugs at 10 µM concentration. Two negative controls were used: 0.1% DMSO and Bicalutamide. 10 % DMSO was used as positive control. Cells were incubated for 24 h. On the day of analysis, 20 µl of Cell Titer Blue reagent (Promega) was added to each well containing 100 µl media. The plate was incubated for 2 h at 37°C at 5% CO_2. Fluorescence was measured using excitation/emission wavelength of 560/590 nm using ClARIOstar Luminescence plate reader (BMG Labtech).

Data were analysed using GraphPad Prism 6.01 (GraphPad Software, Inc, La *J*olla California, USA). One-way Anova analysis was used to perform statistical studies. The Dunnett's multiple

comparison test was used to compare each drug and the positive control to the negative control Bicalutamide.

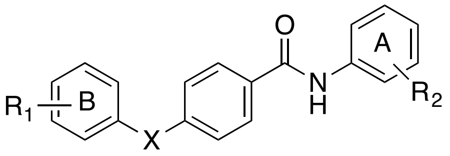

Compound	R_1	X	R_2	Cell Viability		Antiproliferative data
				Mean (%)*	SEM** (±)	Abs. IC_{50} (μM)***
**Bicalutamide**	-	-	-	94.57	5.47	52.42
**10% DMSO**	-	-	-	1.45	0.47	-
**0.1 % DMSO**	-	-	-	100	-	-
**11a**	4-OH	S	4-CN, 3-CF_3	65.66	8.20	7.08
**11b**	4-OH	S	4-NO_2, 3-CF_3	65.39	15.12	4.68
**11c**	4-OH	S	3,5-CF_3	64.19	6.34	**3.28**
**11d**	4-OH	S	2,5-CF_3	53.70	3.95	12.09
**11e**	4-OH	S	4-SF_5	49.45	2.77	5.43
**11f**	4-OH	S	3-SF_5	60.55	9.25	4.01
**11g**	4-OH	S	4-CF_3	66.33	9.68	8.09
**11h**	4-OH	S	3-CF_3	85.59	17.86	7.69
**11i**	4-OH	S	2-CF_3	111.47	8.95	25.25
**12a**	3-CF_3	S	4-CN, 3-CF_3	60.02	1.81	5.50
**12h**	3-CF_3	S	3-CF_3	111.51	8.25	12.47
**13a**	4-OH	SO_2	4-CN, 3-CF_3	72.97	5.99	8.84
**13b**	4-OH	SO_2	4-NO_2, 3-CF_3	82.26	14.09	7.24
**13c**	4-OH	SO_2	3,5-CF_3	65.73	9.65	8.50
**13d**	4-OH	SO_2	2,5-CF_3	n.d	n.d	25.24
**13e**	4-OH	SO_2	4-SF_5	38.35	4.72	5.37
**14e**	4-OH	SO	4-SF_5	59.67	8.97	**3.75**
**13f**	4-OH	SO_2	3-SF_5	76.24	12.87	6.18
**13g**	4-OH	SO_2	4-CF_3	61.77	10.95	19.69
**13h**	4-OH	SO_2	3-CF_3	87.56	13.91	16.84
**13i**	4-OH	SO_2	2-CF_3	128.95	8.59	49.07
**14i**	4-OH	SO	2-CF_3	104.02	23.95	88.19
**15a**	3-CF_3	SO_2	4-CN, 3-CF_3	79.00	8.61	4.71
**15h**	3-CF_3	SO_2	3-CF_3	83.92	11.29	n.d

Table S2. Cell Viability assay at 10 μM. * Mean % of cell viability relative to control; ** Errors are calculated as standard error of the mean; ***Geometric mean.

1.5 References

1. L. Costantino, A.M. Ferrari, M.C. Gamberini and G. Rastelli, *Bioorg. Med. Chem.,* 2002, **10**, 3923-3931.
2. Molecular Operating Environment (MOE 2015.10); Chemical Computing Group, Inc.: Montreal, Quebec, Canada; URL http://www.chemcomp.com .
3. M. Bassetto, S. Ferla, F. Pertusati, S. Kandil, A.D. Westwell, A. Brancale and C. McGuigan, *Eur. J. Med. Chem.*, 2016, **118**, 230-243.
4. O. Korb, T. Stützle and T. E. Exner, *Swarm Intell*. 2007, **1**, 115-134.
5. Oncotest GmbH, Freiburg, Germany. http://www.oncotest.com
6. *J*.R Masters, *J*.A. Thomson, B. Daly-Burns, Y.A. Reid, W.G. Dirks, P. Packer, L.H. To*J*i, T. Ohno, H. Tanabe, C.F. Arlett, L.R. Kelland, M. Harrison, A. Virmani, T.H. Ward, K.L. Ayres and P.G. Debenham, *Proc. Natl. Acad. Sci*., 2001, **14**, 8012-8017.
7. W.G Dirks, S. Faehnrich, I.A. Estella and H.G. Drexler, *ALTEX*., 2005, **2**, 103-109.
8. W.A Dengler, *J*. Schulte, D.P. Berger, R. Mertelsmann and H.H Fiebig, *Anti-Cancer Drugs*, 1995, **6**, 522–532.
9. *J*-H. Zhang, T.D.Y. Chung, T.D.Y and K.R. Oldenburg*, J. Biomol. Screen.* 1999*,* **4**, 67-73.
